# Comparison of gene expression and biotransformation activity of HepaRG cells under static and dynamic culture conditions

**DOI:** 10.1038/s41598-021-89710-6

**Published:** 2021-05-14

**Authors:** Loes P. M. Duivenvoorde, Jochem Louisse, Nicole E. T. Pinckaers, Tien Nguyen, Meike van der Zande

**Affiliations:** Wageningen Food Safety Research, P.O. Box 230, 6700 AE Wageningen, The Netherlands

**Keywords:** Biological techniques, Cell biology

## Abstract

Flow conditions have been shown to be important in improving longevity and functionality of primary hepatocytes, but the impact of flow on HepaRG cells is largely unknown. We studied the expression of genes encoding CYP enzymes and transporter proteins and CYP1 and CYP3A4 activity during 8 weeks of culture in HepaRG cells cultured under static conditions (conventional 24-/96-well plate culture with common bicarbonate/CO_2_ buffering) and under flow conditions in an organ-on-chip (OOC) device. Since the OOC-device is a closed system, bicarbonate/CO_2_ buffering was not possible, requiring application of another buffering agent, such as HEPES. In order to disentangle the effects of HEPES from the effects of flow, we also applied HEPES-supplemented medium in static cultures and studied gene expression and CYP activity. We found that cells cultured under flow conditions in the OOC-device, as well as cells cultured under static conditions with HEPES-supplemented medium, showed more stable gene expression levels. Furthermore, only cells cultured in the OOC-device showed relatively high baseline CYP1 activity, and their gene expression levels of selected CYPs and transporters were most similar to gene expression levels in human primary hepatocytes. However, there was a decrease in baseline CYP3A4 activity under flow conditions compared to HepaRG cells cultured under static conditions. Altogether, the present study shows that HepaRG cells cultured in the OOC-device were more stable than in static cultures, being a promising in vitro model to study hepatoxicity of chemicals upon chronic exposure.

## Introduction

The human hepatoma HepaRG cell line is in use for almost two decades^1^ and represents a robust and reliable in vitro model for chemical biotransformation and toxicity studies. HepaRG cells are more similar to human primary hepatocytes than other hepatoma cells lines regarding, for example, the expression levels of genes involved in chemical biotransformation and transport and the induction of cytochrome P450 (CYP) enzyme activities by various xenobiotics^2–6^. In contrast to primary hepatocytes, HepaRG cells are constantly available, stable for at least 4 weeks and they differentiate into both hepatocytes and biliary epithelial cells^7^. Apart from the advantages, the use of HepaRG cells also has some limitations. Gene expression can also deviate from that in primary hepatocytes as described for several genes, including some genes coding for CYP enzymes and transporters, which also resulted in lower basal CYP activity and chemical transport, respectively^4,8,9^. Furthermore, HepaRG cells may be used for up to 4 weeks after differentiation^9,10^, after which they lose their differentiated status and drug metabolizing capacity^11^. This is far longer than the window of use of primary hepatocytes using conventional culture techniques, but it may still be insufficient to study chemical effects upon long-term (chronic) exposure, as an alternative model to in vivo models that are exposed from several weeks up to years.

Over the years, numerous adaptations in cell culture techniques have been reported to further mimic the complexity of the in vivo liver environment and to stabilize the metabolic function of hepatocytes in vitro. A promising approach to improve functionality and stability of the HepaRG model, possibly even over prolonged culture periods, is to grow the cells under flow conditions in a microfluidic device. Microfluidic culture conditions have been shown to significantly improve metabolic function and viability of hepatocytes, which have high energy needs and largely benefit from the fresh supply of nutrients and oxygen and continuous removal of waste products that is offered by microfluidic conditions [reviewed in^12^]. Fluid flow creates shear stresses on the cells, which depend on the design of the culturing device and the flow speeds that are used. For hepatocytes the shear stress should not exceed the level that is found in the liver sinusoid in vivo, since higher rates tend to decrease hepatocyte functionality^13,14^. When adequately applied, fluid flow has been shown to increase the secretion of albumin and urea and to conserve cytochrome P450 activity in primary hepatocytes compared to cells cultured under static conditions^15–19^.

Although HepaRG cells have been used to create liver-on-chip platforms previously, their behaviour under such dynamic conditions versus conventional static culture conditions and versus human primary hepatocytes has not been extensively studied. Furthermore, the influence of the dynamic conditions on the functional stability of HepaRG cells, even after prolonged culture periods, has not been studied yet. Therefore, the present study aims to investigate the functionality of an in vitro HepaRG organ-on-chip (OOC) liver model up to 8 weeks of culture and to compare it with HepaRG cells cultured under conventional static conditions. For this, the morphology, CYP enzyme activity and inducibility, and gene expression patterns were studied. Additionally, CYP enzyme activities and gene expression patterns were compared with those observed in human primary hepatocytes, as this model is considered the ‘golden standard’ for metabolism and toxicity studies in vitro.

## Results

**Figure 1:**
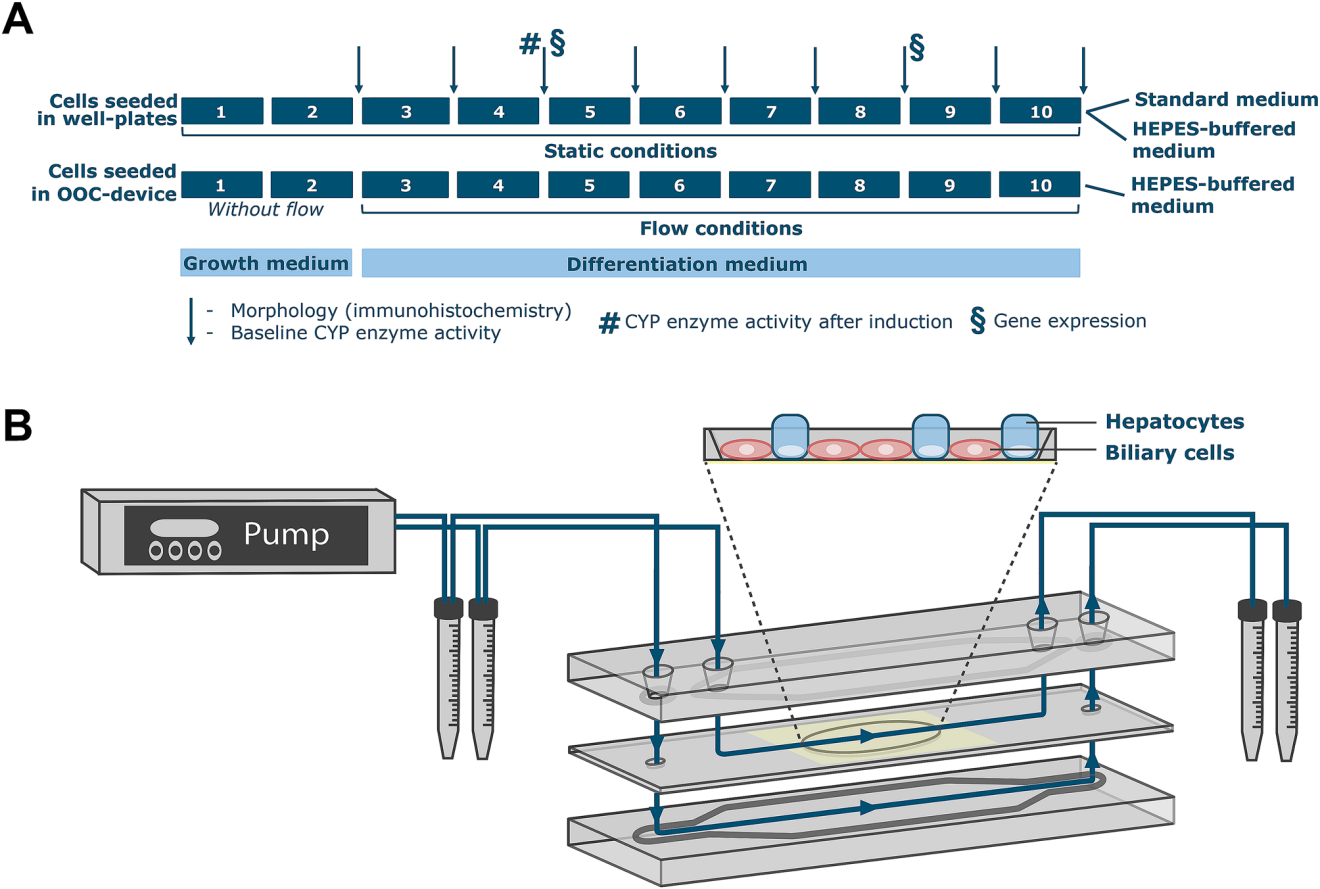
(**A**). Schematic overview of the experimental study design (in weeks). The first 2 weeks after seeding, HepaRG cells were cultured in growth medium (proliferation phase). Afterwards, cells were cultured in differentiation medium for the remaining 8 weeks of the experiment. Cells have been reported to be fully differentiated at the end of cell culture week 4. Arrows indicate when basal CYP enzyme activity was determined and confocal images were made, § when gene expression was measured, and # when CYP activity was induced with TCDD and rifampicin. (**B**) Design of the OOC-device. The chip consists of three glass layers that are pressed together in a chip holder. The silicone gaskets on the upper and lower slide (that are shown in dark grey) form the leak-free boundaries of the apical and basolateral compartment. The blue arrows indicate the flow in and out of the chip. The yellow oval in the middle layer represents the porous membrane on which the cells are grown.

The schematic overview of the experimental setup and the OOC-device used is depicted in Fig. 1A,B. In common cell culture practice, cells are cultured under CO_2_ enriched conditions (i.e. 5%) in an incubator, in which CO_2_ interacts with sodium bicarbonate in the medium as a buffer to keep the pH constant. The OOC-devices however, are closed systems and the medium is thus not exposed to the CO_2_ in the incubator, hampering adequate buffering of the medium. To maintain buffering capacity, HEPES was added to the cell culture medium in the OOC-device. However, HEPES may directly affect the cells, hampering a direct comparison of HepaRG cells in common standard static culture conditions and HepaRG cells in the OOC-device. Therefore, we also evaluated the effect of HEPES on cells grown under static conditions.

### HepaRG cell density

**Figure 2:**
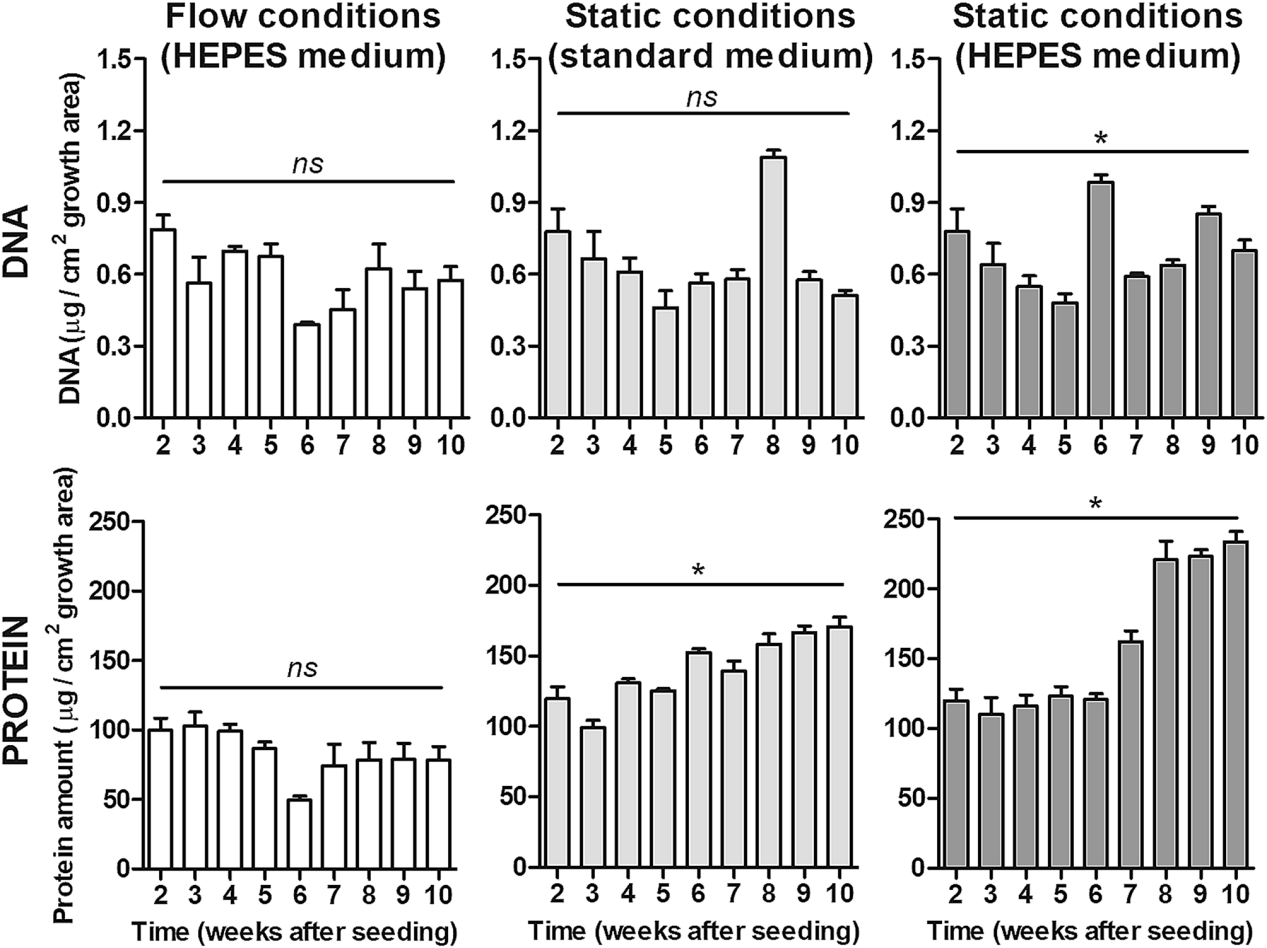
HepaRG cell density as measured as the amount of DNA (**A**) and protein (**B**) per cm^2^ growth area in the OOC-devices (flow conditions; white bars) and in 96-well plates (static conditions; standard medium (light grey bars) and with HEPES medium (dark grey bars)). Differences between weeks within one condition were analyzed with a Kruskal–Wallis test. *ns* not significant, *P < 0.05.

To obtain insight into the proliferation and cell density of HepaRG cells that were grown in the OOC-device and in 96-well plates, we measured DNA and protein contents in cell lysates (Fig. 2). The amount of DNA per cm^2^ of growth area was stable over time, with some fluctuations from week to week, for all conditions (Fig. 2A). The amount of protein was also stable over time in the cells grown under flow conditions, but gradually increased under static conditions (Fig. 2B).

### Morphology

HepaRG cells were cultured in Transwell inserts (static, standard medium) and in the OOC-devices (flow, HEPES medium) to evaluate the morphology of the cells during long-term culturing. Cell confluence and differentiation into distinct cell types were assessed during the 10-week time course of the experiment. Cells were stained using markers for the different cell types (and DAPI to stain cell nuclei) at week 2, 3, 4, 6, 8 and 10. Confocal microscopic imaging revealed that, in both systems and at all time points, HepaRG cells formed a confluent layer that covered the entire membrane (Suppl. Figure 1 and 2). Staining albumin and CK19, which are markers for mature hepatocytes and biliary epithelial‐like cells, respectively, demonstrated the presence of both cell types under both conditions throughout the time course of the experiment. CK19 was detected from week 2 onwards under both culture conditions, whereas albumin was detected from week 3 onwards in the cells grown under flow conditions and from week 4 onwards under static conditions (Suppl. Figure 1). The ratio of hepatocytes to biliary-like cells was around 0.5 under both culture conditions, as expected for differentiated HepaRG cells. In both systems, but especially in the cells in the OOC-device, elaborate CK19-positive structures could be observed, demonstrating interconnectivity between hepatocytes through canaliculus-like structures.

Staining hepatic nuclear factor HNF4α, which is another marker for mature hepatocytes, showed the presence of mature hepatocytes in both systems from week 3 onwards (Suppl. Figure 2). Pgp, which is a transporter located between hepatocytes and bile canaliculi, could be observed from week 2 onwards in both systems. No clear differences were detected in staining intensity or cellular distribution of Pgp between both culture conditions.

### Gene expression

**Figure 3:**
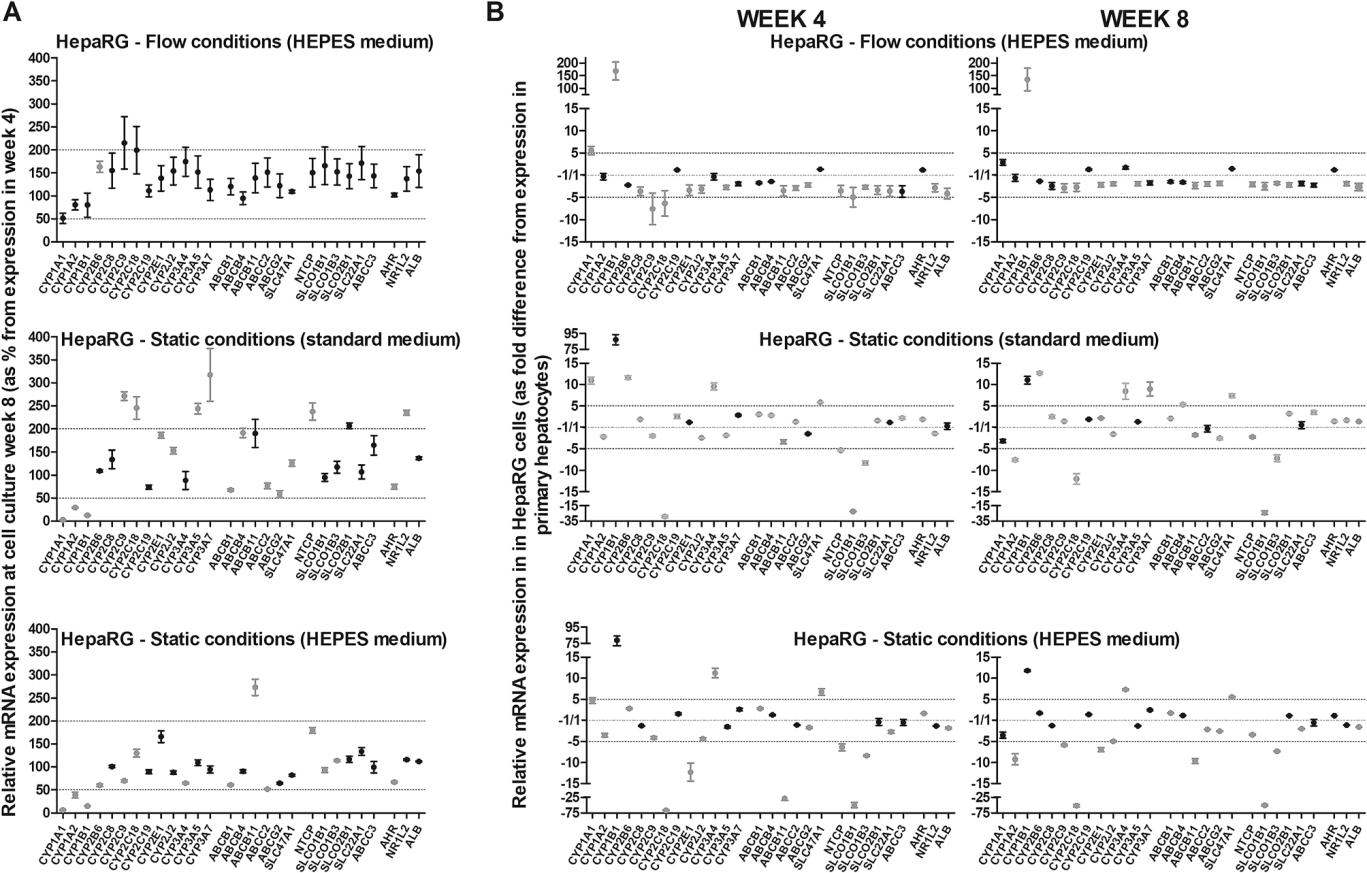
Stability of gene expression (**A**) of the selected genes over a period of 4 weeks of cells grown in the OOC-device (flow conditions) and in 24-well plates (static conditions). Expression at week 8 is given as a percentage of the expression at week 4 (mean ± SEM; n = 3). Symbols in grey are significantly different between week 4 and 8 (unpaired Student’s t-test; *P* < 0.05). Comparison of gene expression in HepaRG cells grown in the OOC-device (flow conditions) and in 24-well plates (static conditions) at week 4 and 8 and in human primary hepatocytes (**B**). Gene expression in HepaRG cells is presented as the fold difference compared to the expression in primary hepatocytes (mean ± SEM; n = 3). Symbols in grey are significantly different between HepaRG cells and primary hepatocytes (One-way ANOVA and Bonferroni post-hoc; *P* < 0.05).

We investigated the influence of different cell culture conditions on the expression of 28 genes involved in chemical biotransformation and transport in differentiated HepaRG cells at 4 and 8 weeks after seeding (Suppl. Table 1). Flow conditions, as well as application of HEPES-buffered medium under static conditions, appeared to have a profound effect on gene expression in HepaRG cells. To facilitate interpretation of the data, we used two approaches; (1) assessment of stability of gene expression over time (Fig. 3A), and (2) comparison of gene expression profiles of HepaRG cells with the ‘golden standard’ primary hepatocytes (Fig. 3B).

#### Stability of gene expression over time

Between week 4 and 8 after seeding HepaRG cells that were cultured under flow conditions appeared to be mostly stable (Fig. 3A). The expression of only one gene (*CYP2B6*; shown in grey) was found to be significantly different between week 4 and 8. Gene expression levels of cells cultured under static conditions using standard medium appeared to be less stable than those in the OOC-device, as the expression levels of 19 of the 28 genes (shown in grey) were significantly different at week 8. Interestingly, gene expression under static conditions appeared more stable when HEPES-buffered medium was used, as here the expression levels of 13 of the 28 genes (shown in grey) were significantly different at week 8.

#### Comparison of gene expression profiles of HepaRG cells with primary hepatocytes

The gene expression profile of HepaRG cells was mostly similar to that of primary hepatocytes when cells were cultured in the OOC-device, although the levels appeared to be generally lower (Fig. 3B). At week 4, expression of 18 genes was significantly different and expression of 15 genes was significantly different at week 8. For most of these differentially expressed genes the expression was approximately two- to three-fold lower compared to primary hepatocytes, and (with the exception of *CYP1B1*, *CYP2C9* and *CYP2C18*) not more than five-fold different. A large difference was observed for *CYP1B1*, for which the expression level in HepaRG cells in the OOC-device was ~ 150 times higher than in primary hepatocytes.

In HepaRG cells cultured under static conditions using standard medium, expression of 23 genes was significantly different from primary hepatocytes at both week 4 and 8. When cells were grown under static conditions with HEPES-buffered medium, expression of 19 and 17 genes was significantly different at week 4 and 8, respectively. In contrast to the HepaRG cells cultured in OOC-devices, no general trend (increase or decrease) in differentially expressed genes was observed in cells cultured under static conditions. Genes showing relatively large differences in expression level include *CYP1B1* (87-fold higher), *CYP2C18* (29-fold lower) and *SLCO1B1* (24-fold lower) in HepaRG cells cultured under static conditions (standard medium); and *CYP1B1* (79-fold higher), *CYP2C18* (67-fold lower), *ABCB11* (29-fold lower) and *SLCO1B1* (51-fold lower) in HepaRG cells cultured under static conditions with HEPES-buffered medium.

### CYP1 and CYP3A4 activity

**Figure 4:**
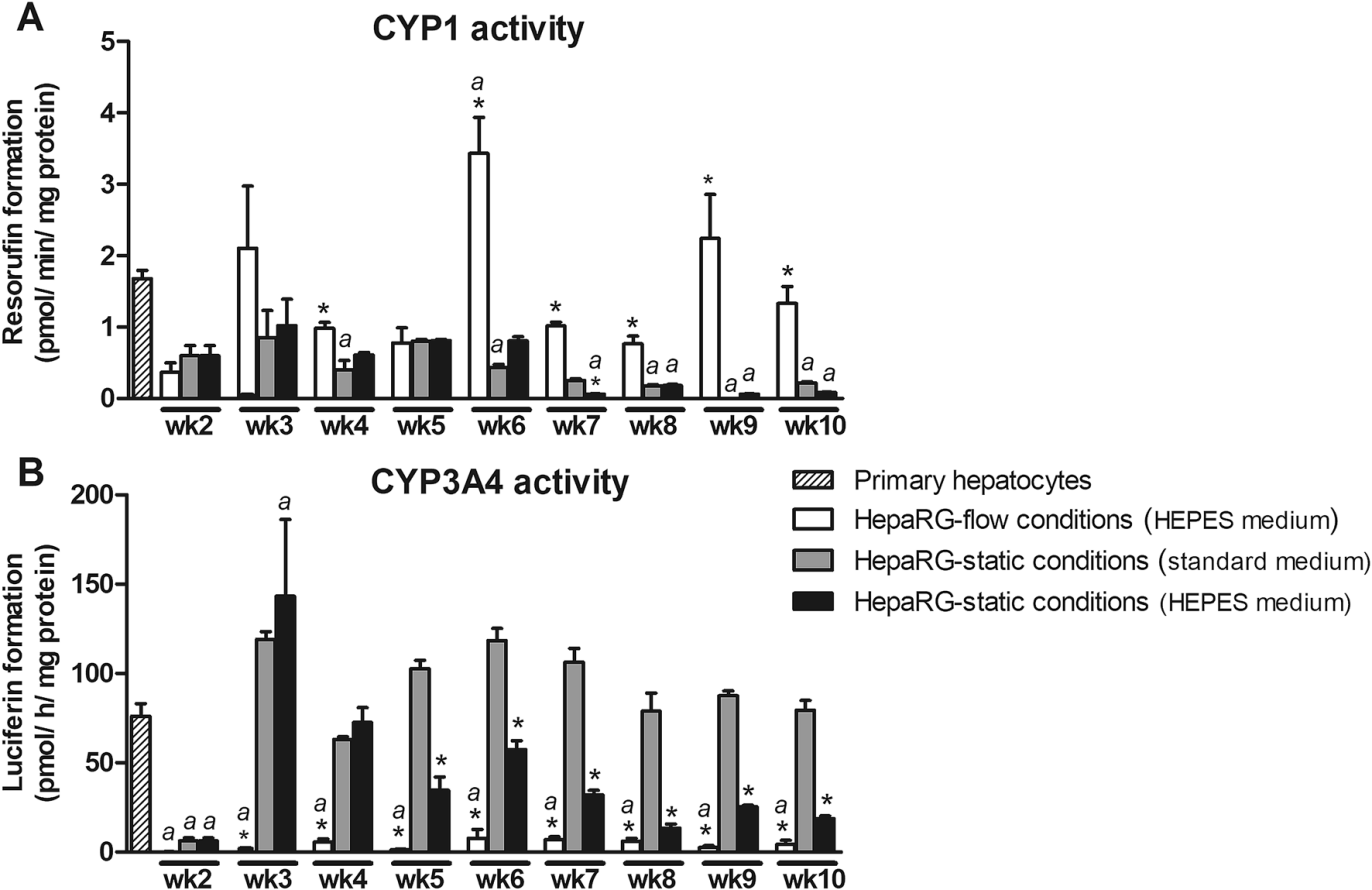
Baseline CYP1 (**A**) and CYP3A4 (**B**) activity in HepaRG cells cultured under flow (OOC device) and static conditions (96-well plates) from week 2 to 10, and in human primary hepatocytes (mean ± SEM; n = 3–6). ^a^Significantly different from primary hepatocytes. *Significantly different from HepaRG static culture with standard medium (comparison within the same week, not assessed for primary hepatocytes). One-way ANOVA and Bonferroni post-hoc tests were used for statistical analyses (*P* < 0.05). Activity was normalized to cell density using protein content data as measured in the cell lysate from the corresponding well/OOC-device. Results of normalization based on DNA content gave similar outcomes (see Suppl. Figure 4).

**Figure 5:**
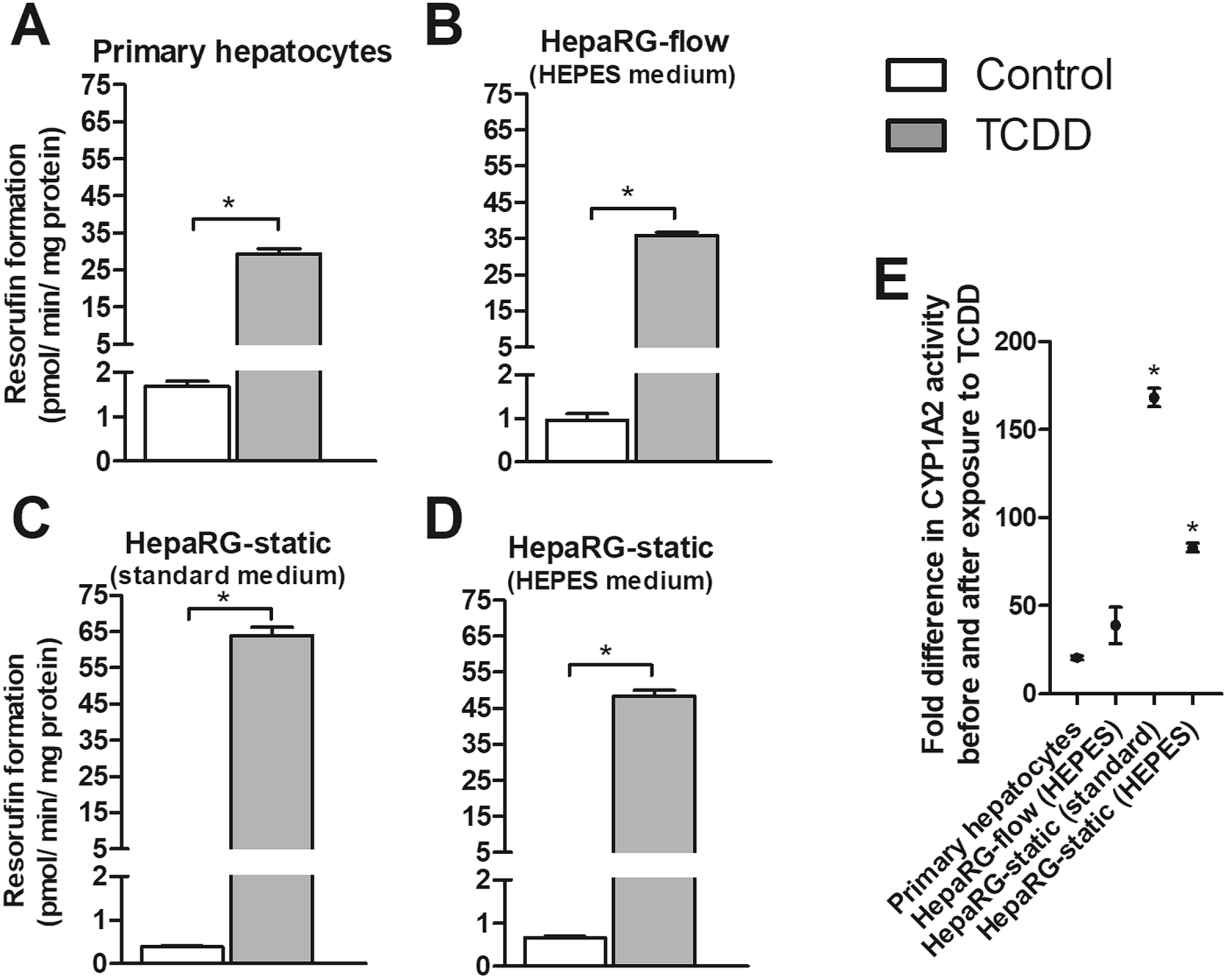
CYP1 activity of primary hepatocytes (**A**) and HepaRG cells (**B**–**D**) before (white bars) and after (grey bars) 48 h exposure to TCDD. HepaRG cells were grown in 24-well plates (static) or in the OOC device (flow) and exposed to TCDD at week 4. *Significant difference with the control (**A**–**D**, unpaired Student's t-test; *P* < 0.05) or with the primary hepatocytes (**E**, One-way ANOVA and Bonferroni; *P* < 0.05). Activity was normalized to cell density using protein content data as measured in the cell lysate from the corresponding well/OOC device.

**Figure 6:**
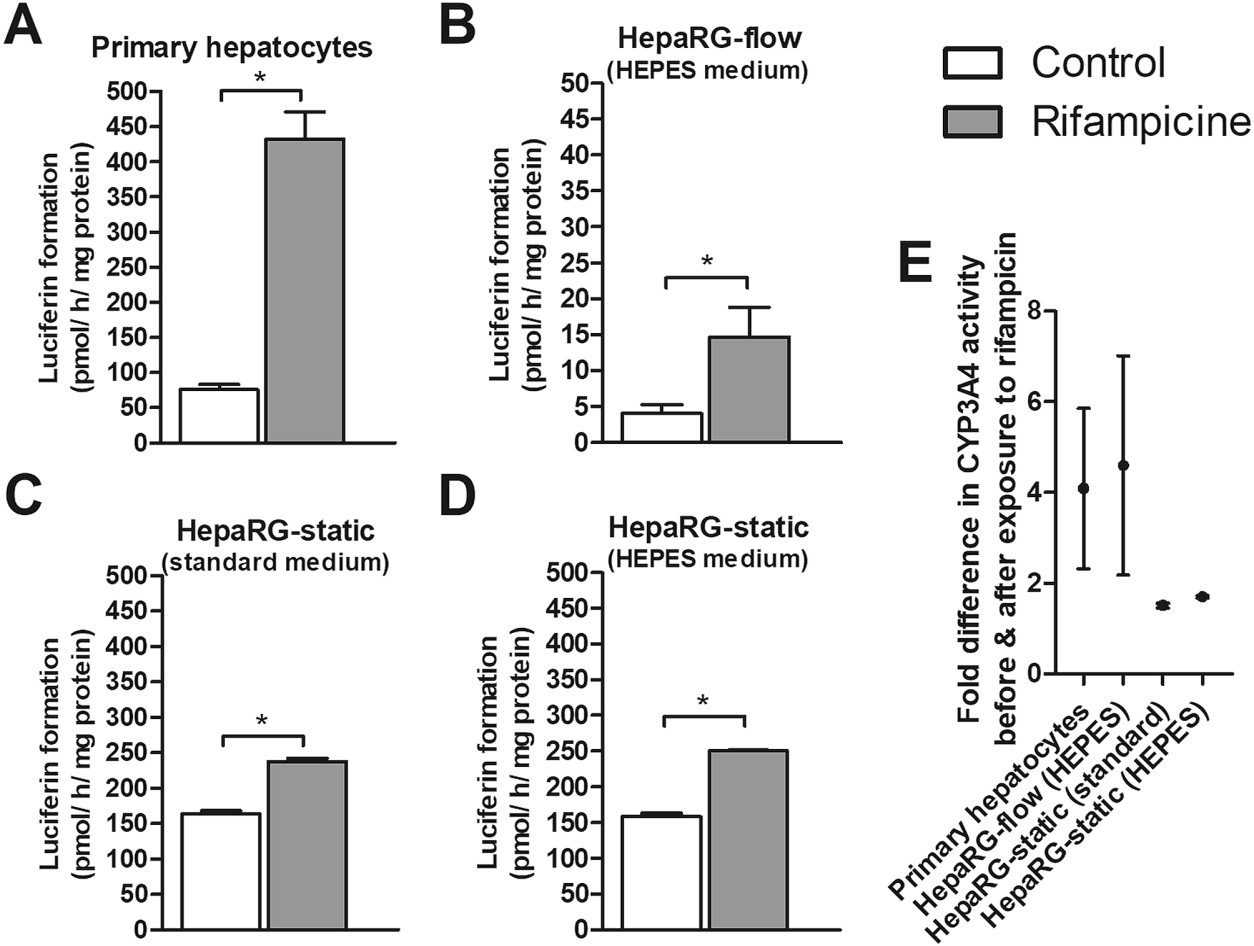
CYP3A4 activity of primary hepatocytes (**A**) and HepaRG cells (**B**–**D**) before (white bars) and after (grey bars) 48 h exposure to rifampicin. HepaRG cells were grown in 24-well plates (static) or in the OOC device (flow) and exposed to rifampicin at week 4. *Significant difference with the control (**A**–**D**, unpaired Student's t-test; *P* < 0.05). Activity was normalized to cell density using protein content data as measured in the cell lysate from the corresponding well/OOC-device.

To obtain more insight into the activity of a selection of CYP enzymes in HepaRG cells cultured under static and flow conditions, the baseline CYP1 and CYP3A4 activity was determined weekly in HepaRG cells (Fig. 4), as well as the activity after induction with a CYP1 and CYP3A4 inducer, respectively (Figs. 5, 6).

#### Baseline CYP activity

CYP1 activity in HepaRG cells under flow conditions was similar to or higher than the activity in primary hepatocytes, whereas CYP1 activity was significantly lower in HepaRG cells cultured under both static conditions, compared to the activity in primary hepatocytes (see Fig. 4A).

Cells grown in the OOC-device had a lower CYP3A4 activity during the complete duration of the experiment compared to the activity in both primary hepatocytes and HepaRG cells grown under static conditions (Fig. 4B). CYP3A4 activity of cells grown under standard conditions was comparable to that of primary hepatocytes, except in week 2 (before full differentiation of the cells) where the CYP3A4 activity was significantly lower. CYP3A4 activity of cells grown under static conditions with HEPES-buffered medium was lower compared to the activity in HepaRG cells under standard conditions.

#### Induction of CYP enzyme activity

Cells were exposed to the CYP1 inducer TCDD and the CYP3A4 inducer rifampicin. CYP1 activity upon a 48 h exposure to 10 nM TCDD and CYP3A4 activity upon a 48 h exposure to 10 µM rifampicin was determined in pooled cryopreserved plateable primary hepatocytes (Figs. 5A, 6A) one day after seeding, and in HepaRG cells (Figs. 5B–D, 6B–D) grown under all culture conditions at week 4. Data were normalized to their respective protein content (data not shown).

TCDD significantly increased CYP1 activity under all culture conditions compared to the controls, showing the largest fold-induction in HepaRG cells grown under standard static conditions (Fig. 5E).

Rifampicin significantly increased CYP3A4 activity in all four culture conditions compared to the controls. Although CYP3A4 activity was very low in HepaRG cells grown in the OOC-device, the fold-induction in CYP3A4 activity (Fig. 6E) was comparable to that in primary hepatocytes: about four-fold, in contrast to the ± two-fold induction in HepaRG cells grown under static conditions.

## Discussion

This study provides insight into the suitability of the OOC model for application in toxicological research, particularly in chronic in vitro toxicity studies. It shows that HepaRG cells cultured in the OOC-device, compared to HepaRG cells under static conditions, have a more stable pattern of gene expression, which was also more similar to that of primary hepatocytes, but that CYP3A4 activity was relatively low.

HepaRG cells are bipotent progenitor cells that fully differentiate into functional hepatocytes and biliary-like cells^7^. In our study, we monitored the morphology of the cells for 8 weeks after the proliferation phase. Confocal imaging showed that under both flow and static conditions, HepaRG cells retained their differentiated morphology until the end of the study. The cell layer remained confluent and the ratio of hepatocytes to biliary cells remained equal. Stability in cell number over time was also demonstrated by the stable DNA content in both systems over time. In contrast, the protein content increased over time under static conditions, while it remained constant under flow conditions. The difference in protein content between both systems might indicate that protein synthesis, protein deposition or protein wash-out is affected by the flow, rather than changes in cell number, given that the DNA content remains the same.

Although we did not attempt to quantify differences in intensity of the immunocytochemical stainings between both conditions, some minor differences were observed. Albumin was detected in week 3 under flow conditions versus in week 4 under static conditions. This might imply that flow conditions accelerate the maturation of hepatocytes, which was also reported in a study by Ong et al.^20^, where flow enhanced differentiation of HepaRG progenitor cells into hepatocytes, or that flow conditions stimulate albumin production by hepatocytes, which is consistent with previous research on the influence of flow on albumin secretion by primary hepatocytes^21–23^.

Flow conditions mimic the in vivo tissue physiology in terms of shear stress, continuous supply of nutrients and oxygen, and continuous removal of waste products. In the present study, HepaRG cells under flow conditions showed to be stable during the 8 week study period in terms of gene expression and CYP enzyme activity. In contrast, in statically grown HepaRG cells, more than two third of the genes of interest were significantly altered over time, pointing to a less robust system. The largest changes in gene expression between week 4 and 8 were found in the expression of *CYP1A1* and *CYP1B1* that decreased 34- and eight-fold in cells under standard static conditions, respectively. The decrease in *CYP1* expression was consistent with the observed decrease in CYP1 activity under static conditions. The results are in agreement with other studies reporting fluctuations in gene expression levels over time in differentiated HepaRG cells cultured in 24- and 96-well plates^2,4^. However, gene expression of a number of selected genes^10^ and CYP enzyme activity^9^ were also shown to remain stable between cell culture week 4 and 8 when HepaRG cells were cultured in 6-well plates. The 6-well-plates have a larger surface-to-volume-ratio than the 24-well plates that were used in our study for gene expression analyses, promoting gas exchange with the environment and possibly preventing shortage of oxygen availability at the cellular level. This may relate to a less stable culture in 96- and 24-well plates compared to 6-well plates and may indirectly correspond with the positive effects of flow conditions where oxygen supply is constant. For drug metabolism and cytotoxicity studies the smaller 24- and 96-well plate format, however, is commonly used to increase throughput [e.g.^24,25^].

Limited oxygen supply in static in vitro culture conditions has been reported to increase glycolytic activity in hepatocytes and may lead to triglyceride accumulation^26^, whereas the relatively higher oxygen supply in flow systems reduces intracellular lipid accumulation^27^. At the molecular level, hepatic lipid accumulation is characterized by an increase in the expression of *CYP2E1* both in vivo [reviewed by^28^] and in vitro^29,30^. Also in our studies, *CYP2E1* was 3.2-fold higher under standard static vs. flow conditions and further increased (1.9-fold) in time.

Flow conditions appear to provide better growth conditions due to a constant supply of nutrients and oxygen and the continuous removal of cellular products. It should be noted that we had to use HEPES as a buffering agent in the medium for the flow conditions, as common bicarbonate/CO_2_ buffering cannot be applied in the closed OOC-device. Since the addition of HEPES also had a significant effect on HepaRG stability in static cultures, increased cellular stability in the OOC-device cannot be solely attributed to application of flow, but has to be regarded as an effect of the combined application of flow and HEPES-supplemented medium. Interestingly, the gene expression profile under flow conditions appeared to be most similar to that of primary hepatocytes, indicating that application of flow conditions in the HepaRG cell culture appears to improve the HepaRG cell model as a test system compared to application of static culture conditions, and is suggested to be a good model for chronic in vitro toxicity studies.

Besides gene expression we also studied the activity of CYP1 and CYP3A4 as examples of important chemical biotransformation enzymes. HepaRG cells grown under flow conditions had, compared to cells grown under standard static conditions, a high CYP1 and a low CYP3A4 baseline activity. Consistently, HepaRG cells under flow conditions had high *CYP1A1*, *CYP1A2* and *CYP1B1* and low *CYP3A4* baseline mRNA expression, suggesting that the changes in gene expression translate to the observed activity. The increase in CYP1 activity under flow conditions is in line with previous research that shows that the enzyme activity in hepatocytes increases under flow^31,32^. The decrease in CYP3A4 activity under flow is also consistent with previous studies that show that CYP3A4 enzyme activity is especially sensitive to shear stress. A two-fold increase in fluid flow shear was, in fact, found to reduce CYP3A4 activity more than two-fold, whereas it doubled CYP1 activity in human primary hepatocytes^32^. This indicates that mechanical stimulation of cells, like shear stress stimulation, can negatively affect CYP activity, besides the positive effects caused by continuous oxygenation, nutrient supply and waste removal. However, contradicting results have been reported on the direction of regulation of CYP enzyme activity due to shear stress. Inhibitory effects are generally attributed to shear stress levels exceeding levels reported in vivo, causing a reduction in cell function. High shear stresses in in vitro experiments were described to reduce hepatic metabolic activity^33^, albumin and urea synthesis rates^13^, and transcription of *CYP3A4*^14^. Shear stresses at the surface level of human liver sinusoids are around 0.1–0.5 dyne/cm^2^ ^34,35^ and shear stresses at the surface level of hepatocytes are expected to be several orders of magnitude lower^36^. In our study, cells were constantly exposed to a shear stress rate of ∼ 0.0002 dyne per cm^2^, which possibly affected CYP3A4 baseline mRNA expression and related enzyme activity. Evaluating lower shear stresses is therefore recommended for future research. It is, however, important to notice that, despite the low baseline activity, CYP3A4 activity of HepaRG cells under flow conditions was still inducible by rifampicin to a similar extent (± four-fold induction) as that observed in primary hepatocytes. This implies that HepaRG cells cultured under flow conditions adequately respond to CYP-inducing agents.

To the best of our knowledge, this is the first study that characterizes HepaRG cells cultured under flow conditions and conventional static conditions for a prolonged period of 8 weeks after the proliferation phase. Flow conditions improved HepaRG stability at the molecular and functional level, although the CYP3A4 baseline activity was significantly reduced. Furthermore, culturing HepaRG cells in the OOC-device with flow made the cells behave more similar to human primary hepatocytes at the gene expression level for a selection of CYP enzymes and transporter proteins. For future studies it would be of interest to further study the metabolic capacity of the OOC HepaRG model using different model compounds. Altogether, the present study shows that HepaRG cells cultured in the OOC-device are a promising in vitro model to study hepatoxicity of chemicals upon chronic exposure.

## Materials and methods

### Organ-on-chip design

The microfluidic organ-on-a-chip (OOC) device (Micronit, Enschede, the Netherlands) has been previously described^37^. In short, the device (Fig. 1B) consists of two glass flow chambers that are separated by a middle layer with a microporous polyester cell culture membrane (0.4 μm pore size, 12 μm thickness, 1.6 × 10^6^ pore density and 1 cm^2^ surface area). The flow through the apical and basolateral chambers was kept constant at 100 μl/h using an air-pressured microfluidic mass flow control unit (Fluigent, Jena, Germany), resulting in a shear stress rate at the surface of the membrane in the apical compartment of ∼ 0.0002 dyne per cm^2^ ^38^.

The setup used consists of four air-pressurized pumps that control the flow of 16 OOC-devices individually, placed in an incubator at 37 °C. Cell culture medium is supplied and collected separately to and from the apical and basolateral chamber through PEEK and PTFE tubing (Idex Health&Science, Oak Harbor, USA).

### HepaRG cell culture

The human hepatocellular carcinoma cells (HepaRG; Biopredic International, Rennes, France) were cultured as described previously^24^. In short, after seeding, cells were kept in growth medium consisting of William's Medium E + GlutaMAX (ThemoFisher Scientific, Landsmeer, The Netherlands) supplemented with 10% Good Forte filtrated fetal bovine serum (FBS; PAN Biotech, Aidenbach, Germany), 1% Pen-Strep (100 U/mL penicillin, 100 μg/mL streptomycin; Capricorn Scientific, Ebsdorfergrund, Germany), 50 μM hydrocortisone hemisuccinate (sodium salt) (Sigma-Aldrich), and 5 μg/mL human insulin (PAN Biotech) for 2 weeks (proliferation phase). Under static culture conditions, medium was refreshed every 2–3 days.

For static culture conditions, cells were seeded in black coated 96-well plates (Greiner Bio-One, Frickenhausen, Germany; 9000 cells per well in 100 μL) for CYP activity under baseline conditions; in transparent 24-well plates (Greiner Bio-One; 55,000 cells per well in 500 μL) for RNA isolation and CYP induction studies; or in Transwell inserts (Corning; 25,000 cells per insert in 250 μL in the apical compartment (750 μL in the basolateral compartment); 0.4 μm pore 12 mm polyester membrane) for immunocytochemistry. For flow culture conditions, cells were seeded in the OOC-device on the polyester membrane of the middle layer (25,000 cells per chip in 250 μL). The first 2 weeks after seeding (during proliferation), cells were maintained on the membrane without flow. Polyethyleentereftalaat (PET) membranes in the OOC-device and Transwell inserts were coated with human collagen type I from fibroblasts (Sigma-Aldrich; 10 µg/membrane).

After the 2 weeks on growth medium, cells were cultured for two days in growth medium supplemented with 0.85% DMSO to induce differentiation. Subsequently, cells were cultured for 12 days in growth medium supplemented with 1.7% DMSO (differentiation medium) to complete differentiation. At this stage, the cells grown in the OOC device were cultured under flow conditions (using a flow of 100 µL/h). After the 2-week differentiation phase, all cells (under static and flow conditions) were kept for an additional 6 weeks in differentiation medium (also see Fig. 1A for the experimental design).

Cells that were cultured under static conditions using standard medium, referred to as ‘static conditions (standard medium)’, were maintained in an incubator (humidified atmosphere with 5% CO_2_ at 37 °C). Cells that were cultured in the OOC-device were maintained at 37 °C and at ambient CO_2_ conditions, as the OOC-device (including its medium reservoirs) is a closed system and medium cannot be buffered using CO_2_ in the incubator. At ambient CO_2_ conditions the pH of standard cell culture medium increases to non-physiological toxic levels. Therefore, the concentration of sodium bicarbonate in the medium was reduced from 2.2 to 0.84 g/L and 25 mM HEPES (Sigma-Aldrich) was added to offer adequate buffering capacity^37^. These conditions are referred to as ‘flow conditions (HEPES medium)’. To assess the influence of the change in cell culture medium, HepaRG cells were also cultured under static conditions using the same HEPES-buffered medium as used in the OOC devices in a humidified incubator at 37 °C at ambient CO_2_ conditions. These conditions are referred to as ‘static conditions (HEPES medium)’.

### Human primary hepatocyte cell culture

Pooled cryopreserved plateable primary human hepatocytes (5-donor pool; Thermo Fisher Scientific) were, according to the manufacturer’s instructions, seeded in collagen I-coated transparent 24-well plates (Thermo Fisher Scientific) at 400,000 cells in 500 μL plating medium (William’s E medium, supplemented with 5% FBS, 1 µM dexamethasone, 1% penicillin–streptomycin, 4 µg/mL human recombinant insulin, 2 mM GlutaMAX and 15 mM HEPES) per well. Four hours after seeding, cells were coated with Geltrex Matrix (Thermo Fisher Scientific) at 0.35 mg/mL and medium was replaced with 500 μL maintenance medium (William’s E medium, supplemented with 0.1 µM dexamethasone, 0.5% penicillin–streptomycin, 6.25 µg/mL human recombinant insulin, 1.25 mg/mL bovine serum albumin, 2 mM GlutaMAX and 15 mM HEPES). Maintenance medium was refreshed daily.

### Determination of cell density in well plates and OOC-device

The cell density (HepaRG cells and primary hepatocytes) was determined to normalise CYP enzyme activity data and to monitor cell viability during the experiment. Cells were harvested and lysed in 50 µL (96-well plate), 190 µL (24-well plate) or 100 µL (OOC-device) cold RIPA buffer (Sigma-Aldrich). The cell lysate was stored at − 80 °C and later used to determine protein content using a Pierce BCA Protein colorimetric assay kit (Thermo Fisher Scientific) and to determine dsDNA content using a Quant-iT PicoGreen dsDNA Assay Kit (Thermo Fisher Scientific). Both assays were performed according to the manufacturer’s protocol.

### Immunocytochemistry and confocal microscopy

Immunocytochemical stainings for hepatocyte nuclear factor 4 (HNF4), albumin, keratin 19 (KRT19, or CK19) and P-glycoprotein (ABCB1, or Pgp) were performed at week 2, 3, 4, 6, 8 and 10 (after start cell culture) in HepaRG cell cultures that were grown under static conditions (standard medium in Transwell inserts) or under flow conditions (HEPES medium in the OOC-device). HNF4 and albumin are expressed in hepatocytes in the nucleus and cytoplasm, respectively, KRT19 is expressed in biliary epithelial‐like cells and Pgp is a transporter located between hepatocytes and bile canaliculi. Cell culture medium was removed and cells were washed with Phosphate Buffered Saline (PBS) and fixated with 3.7% formaldehyde in PBS for 10 min. Cells were then washed in PBS and permeabilized with 0.25% Triton X100 in PBS for 10 min (all chemicals from Sigma-Aldrich). To prevent unspecific binding, cells were blocked in 5% normal donkey serum and 1% serum bovine albumin in PBS for 30 min, after which antibody incubation started: Rabbit monoclonal anti-HNF4α (Abcam, 1:250 dilution for 1 h), Goat polyclonal anti-albumin (Abcam, 1:200 dilution for 1 h), Mouse monoclonal anti-CK19 (Thermo Fisher Scientific, 1:200 dilution for 1 h) and Mouse monoclonal anti-Pgp (Abcam, 1:200 dilution for 1 h). Antibodies were labelled with the following secondary antibodies (all from Abcam and 1 h incubation with 1:200 dilution): Goat-anti-Rabbit Alexa-594, Donkey-anti-Mouse Alexa-488 and Donkey-anti-Goat Alexa-594. Nuclei were visualised with DAPI diluted 1:1000 in PBS. All steps were performed at room temperature and with intermediate washing steps with PBS. The PET membrane with cells was then cut from the Transwell insert or OOC-device middle layer with a razor blade and embedded on a microscope slide using Diamond anti-fade mountant (Invitrogen) and a spacer (0.12 mm thickness; Grace Bio-Labs, Bend, USA).

The cells were analysed as reported recently^37^ using a confocal microscope (LSM 510 UVMETA; Carl Zeiss, Germany). Samples were excited with 405, 488 and 543 nm lasers. Multi-tracked images were captured to avoid bleed through. The used pinholes were in the range of 70–78 µm at magnification 40. The gain and offset for the different channels were kept constant during the entire experiment.

### RNA isolation and real-time qPCR

Total RNA was isolated from HepaRG cells grown in 24-well plates and in the OOC-device (at 4 and 8 weeks after seeding) and from primary hepatocytes grown in 24-well plates (72 h after seeding) with the RNAeasy kit (Qiagen, Venlo, the Netherlands) according to the manufacturer’s instructions. In short, RLT-buffer (provided in the kit) was supplemented with 1% β-mercapthoethanol and used to lyse the cells. Cell lysates were homogenized and purified with QIA shredder columns (Qiagen) and the buffers and columns provided in the kit. DNA was removed by a 15 min incubation with the RNAse-free DNAse set (Qiagen). RNA concentration and purity were measured using a Nanodrop (IsoGen Life Science, Maarssen, The Netherlands). RNA of individual samples (three samples per condition) was used for cDNA synthesis using the iScript cDNA synthesis kit (Bio-Rad). qRT-PCRs were performed with Sensimix SYBR No-ROX Mix (Bioline, London, UK) using the C100 Touch Thermal cycler CFX384 (Bio-Rad). A standard curve using serial dilutions of pooled samples (cDNA from all samples) was taken along with every assay. Only standard curves with an efficiency between 90 and 110% and a correlation coefficient above 0.99 were accepted. Individual samples were measured in duplicate (technical replicates). Data were normalized against the geometrical mean of three reference genes, namely acidic ribosomal phosphoprotein P0 (*36B4*), glyceraldehyde-3-phosphate dehydrogenase (*GAPDH*) and ribosomal protein L27 (*RPL27*), which had a stable expression between culture conditions and the two cell types (see suppl. Figure 3). To evaluate the stability of the genes over time, the relative expression of each gene at week 8 was calculated as a percentage of the relative expression at week 4. For the comparison of gene expression data of the HepaRG cells with the primary hepatocytes, relative mRNA expression was used to calculate the fold-difference between pooled primary human hepatocytes and HepaRG cells. Results are based on three biological replicates per condition per time point. Primers (see suppl. table 2) were designed using the NCBI Primer-Blast (NCBI website).

### Analysis of P450 enzyme activity

CYP1 enzyme activity was determined in HepaRG cells and primary hepatocytes using an EROD assay^39^. For this assay, cells were exposed to 5 µM ethoxyresorufin (Sigma-Aldrich) in cell culture medium without phenol red. Ethoxyresorufin is converted to resorufin by the enzymes CYP1A1, CYP1A2 and CYP1B1^40^, which is hereafter referred to as CYP1 activity. Cell culture medium was collected after one hour exposure to ethoxyresorufin and the fluorescence of the formed resorufin was detected at excitation 510 nm and emission 586 nm with a micro-plate reader (Biotek, Winooski, USA). Baseline CYP1 activity was determined weekly in HepaRG cells between week 2 and 10 of culture. CYP1 enzyme activity was also determined before and after exposure to TCDD in HepaRG cells and primary hepatocytes (for time points and exposure conditions see next section). The volumes of ethoxyresorufin added to the well plates and OOC-devices were as follows: 75 µL/well for cells grown in 96-well plates, 285 µL/well for cells grown in 24-well plates and 150 µL/chip for cells grown in OOC-devices. Medium samples were stored at − 80 °C and measured together in black polystyrene, non-binding 96-well plates (Greiner Bio-one). All samples were corrected for the background fluorescence of the exposure medium (5 µM ethoxyresorufin).

CYP3A4 activity was determined in HepaRG cells and primary hepatocytes using a CYP3A4 Assay Luciferin-IPA assay kit (Promega, Madison, USA), which mainly monitors CYP3A4 activity with minor of contributions of CYP3A5 and CYP3A7. For this, cells were exposed to 3 µM luciferin-IPA in cell culture medium without phenol red for one hour. CYP3A4 converts the substrate luciferin isopropyl acetal (IPA) to luciferin. After one hour exposure to luciferin-IPA, cell culture medium was collected and incubated for 20 min with a Luciferin Detection Reagent (provided in the kit). Formed luciferin was detected as a luminescent signal with the microplate reader (Biotek). Baseline CYP3A4 enzyme activity was determined weekly in HepaRG cells between week 2 and 10 of culture. CYP3A4 enzyme activity was also determined before and after exposure to rifampicin in HepaRG cells and primary hepatocytes (for time points and exposure conditions see next section). The volumes of luciferin-IPA added to the well plates and OOC-devices were as follows: 50 µL/well for cells grown in 96-well plates, 150 µL/well for cells grown in 24-well plates and 100 µL/chip for cells grown in the OOC-device. Medium samples were stored at − 80 °C and measured together in white polystyrene, non-binding 96-well plates (Greiner Bio-one). All samples were corrected for the background fluorescence of exposure medium (3 µM luciferin-IPA).

Directly after performing the CYP enzyme activity assays, the cells were lysed and collected in RIPA lysis buffer (Thermo Fisher Scientific) and the protein and DNA content of the cell lysate was determined and used to normalize the CYP enzyme activity data.

To determine baseline CYP enzyme activity, 6 wells per 96-well plate, of which two wells were pooled into one sample (n = 3) were used for the cells under static conditions, and 3 to 4 OOC-devices (n = 3/4) were used for cells under flow conditions.

### TCDD and rifampicin exposure for CYP induction studies

HepaRG cells (grown in 24-well plates and in the-OOC-device) were exposed for 48 h to 10 nM TCCD or 10 µM rifampicin at cell culture week 4. Primary hepatocytes were exposed to TCCD or rifampicin one day after seeding. CYP enzyme activity was measured just prior to TCDD/rifampicin exposure and directly after the exposure (48 h) in the same wells/chips (n = 3). Cells were exposed to 10 µM rifampicin or to 10 nM TCDD for 48 h in 500 µL cell culture medium per well and 300 µL cell culture medium per OOC-device. The cells on the OOC-device remained under static conditions during the exposure. Exposure medium was refreshed after 24 h. Directly after performing the CYP enzyme activity assays, the cells were lysed and collected in RIPA lysis buffer (Thermo Fisher Scientific) and the protein content of the cell lysate was determined and used to normalize the CYP enzyme activity data.

### Statistical analysis

Data are expressed as mean ± SEM. Statistical analyses were performed using GraphPad Prism (Graphpad Software, San Diego, USA). Results were checked for normality using the Kolmogorov–Smirnov normality test. Measurements at single time points between two groups were analyzed by unpaired Student’s t-tests. Measurements at single time points among multiple groups were analyzed by One-way ANOVA and Bonferroni post hoc analysis or with the Kruskal–Wallis test and Dunns post-hoc test. *P* < 0.05 was considered statistically significant.

## Supplementary information

Supplementary Informations.
