# Supplementary material for: Comparison of gene expression and biotransformation activity of HepaRG cells under static and dynamic culture conditions
Source: Sci Rep. 2021 May 14;11:10327. doi: 10.1038/s41598-021-89710-6 (PMC8121841; doi:10.1038/s41598-021-89710-6)
Supplement: Supplementary file 1 — Supplementary Informations. [file 41598_2021_89710_MOESM1_ESM.pdf]

Comparison of gene expression and biotransformation activity of HepaRG cells under static and dynamic culture conditions

Authors
Loes PM Duivenvoorde*, Jochem Louisse, Nicole ET Pinckaers, Tien Nguyen and Meike van der Zande

Author affiliations
Wageningen Food Safety Research, P.O. Box 230, 6700 AE Wageningen, The Netherlands
*Correspondence to Loes.Duivenvoorde@wur.nl

Supplementary information

Supplementary figure 1

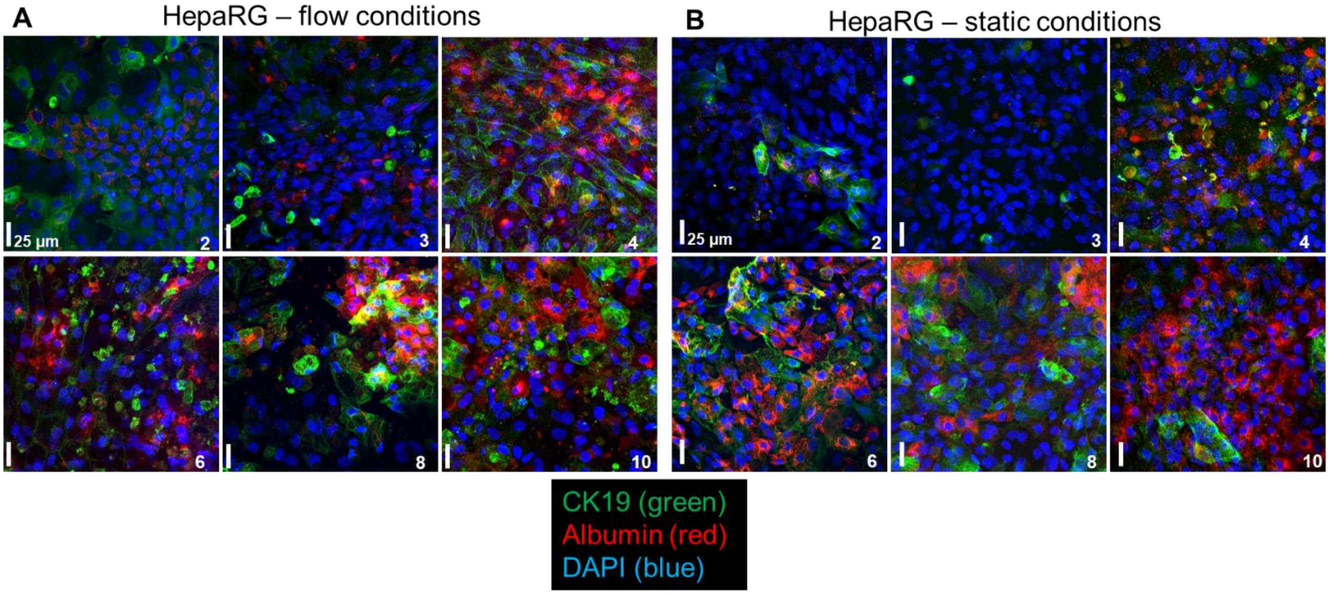

Representative confocal microscopy images of a KRT19 (CK19) and albumin staining in HepaRG cells cultured under flow conditions (A) or static conditions (B) at week 2, 3, 4, 6, 8 and 10. The presence of albumin (red) indicates mature hepatocytes, whereas KRT19 (green) is only present in biliary epithelial cells. Nuclei were counterstained with DAPI (blue). The scale bar represents 25 µm, all pictures are from the same magnification.

Supplementary figure 2

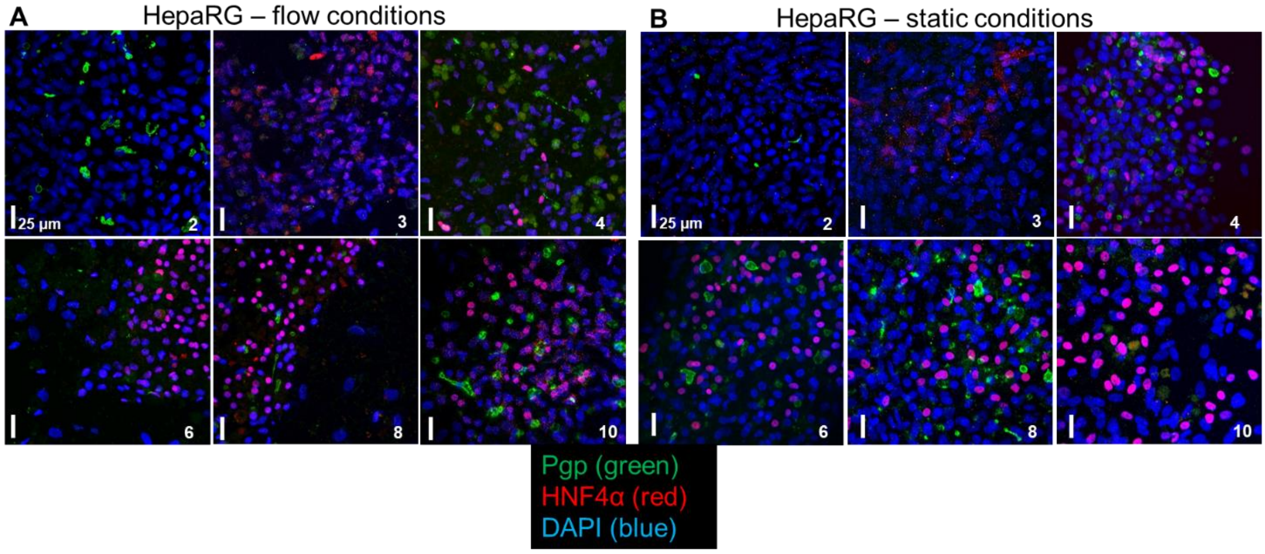

Representative confocal microscopy images of a Pgp and HNF4α staining in HepaRG cells cultured under flow conditions (A) or static conditions (B) at week 2, 3, 4, 6, 8 and 10. The presence of the nuclear factor HNF4α (red) indicates mature hepatocytes, whereas Pgp (green) is present as a transporter in the membrane of bile canaliculi. Nuclei were

counterstained with DAPI (blue). The scale bar represents 25 µm, all pictures are from the same magnification.

Supplementary figure 3

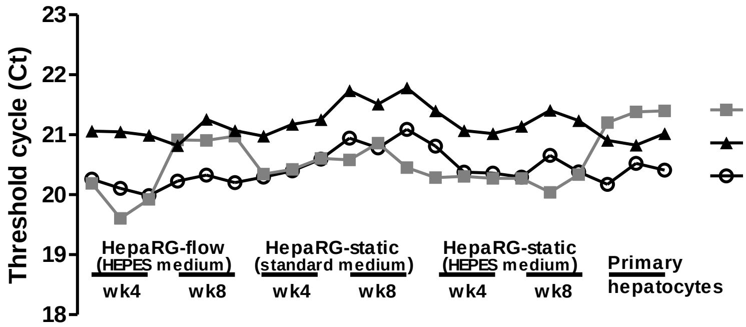

Expression level of the housekeeping genes 36B4, GAPDH and RPL27 that were used to calculate the relative expression of the genes of interest as measured with real-time reverse transcription-PCR (RT-qPCR). Expression levels of individual samples (three per condition) are shown as Ct-values (threshold cycle), which is the intersection between the amplification curve and a threshold line.

Supplementary figure 4

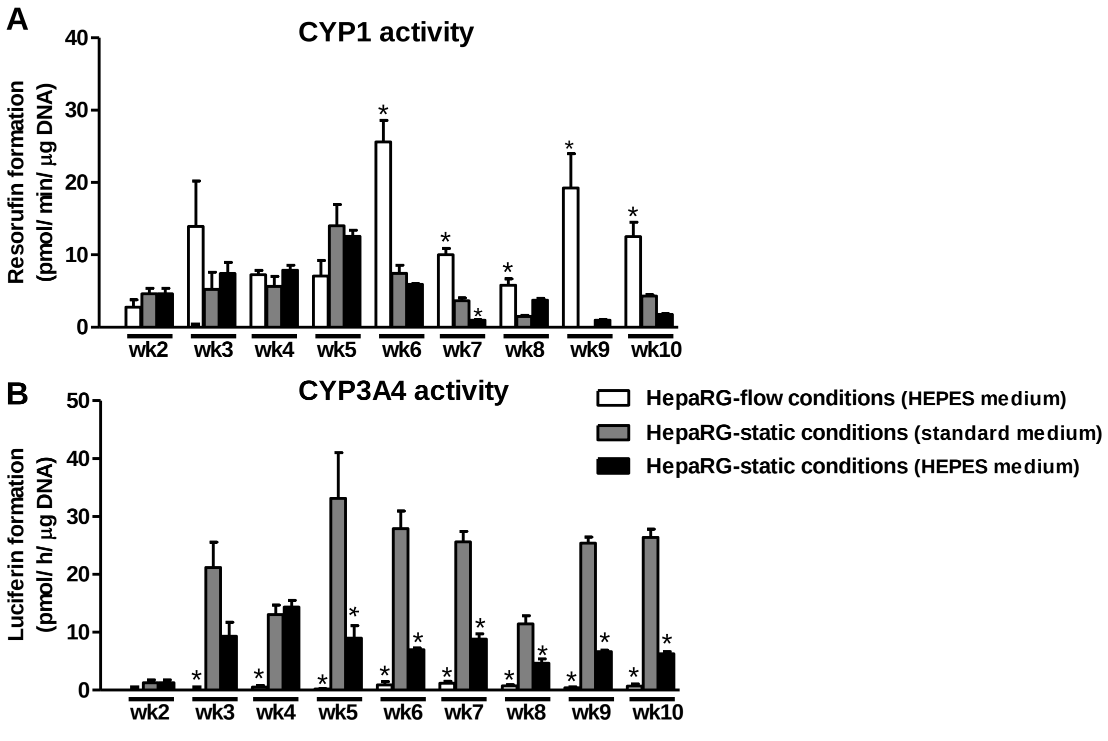

Baseline CYP1 (A) and CYP3A4 (B) activity in HepaRG cells cultured under flow and static conditions during week 2 to 10 (mean ± SEM; n=3-6). Data were corrected for the amount of dsDNA in the cell lysate. Significantly different from HepaRG static culture with standard medium. One-way ANOVA and Bonferroni post-hoc tests were used for statistical analyses (P<0.05).

Supplementary table 1 *Relative mRNA expression of HepaRG cells and primary hepatocytes*

		Average expression	Standard deviation	Significance (comparison to prim. hep.)	Significance (comparison to HepaRG static (standard) within wk4 or wk8)
	**Cytochrome P450 enzymes**				
**CYP1A1**	HepaRG flow (HEPES) wk4	1.477	0.4377	***	*
	HepaRG flow (HEPES) wk8	0.762	0.2881	ns	*
	HepaRG static (standard) wk4	2.900	0.3824	****	
	HepaRG static (standard) wk8	0.085	0.0174	ns	
	HepaRG static (HEPES) wk4	1.251	0.3147	**	**
	HepaRG static (HEPES) wk8	0.080	0.0248	ns	ns
	Primary human hepatocytes	0.266	0.0420		
**CYP1A2**	HepaRG flow (HEPES) wk4	1.953	0.2390	ns	**
	HepaRG flow (HEPES) wk8	1.576	0.3744	ns	**
	HepaRG static (standard) wk4	0.867	0.1027	***	
	HepaRG static (standard) wk8	0.255	0.0214	****	
	HepaRG static (HEPES) wk4	0.557	0.1332	****	*
	HepaRG static (HEPES) wk8	0.217	0.0617	****	ns
	Primary human hepatocytes	1.914	0.2745		
**CYP1B1**	HepaRG flow (HEPES) wk4	2.421	0.8943	***	ns
	HepaRG flow (HEPES) wk8	1.940	1.1057	**	*
	HepaRG static (standard) wk4	1.255	0.1608	ns	
	HepaRG static (standard) wk8	0.159	0.0225	ns	
	HepaRG static (HEPES) wk4	1.132	0.1310	ns	ns
	HepaRG static (HEPES) wk8	0.170	0.0049	ns	ns
	Primary human hepatocytes	0.014	0.0017		
**CYP2B6**	HepaRG flow (HEPES) wk4	0.124	0.0103	ns	****
	HepaRG flow (HEPES) wk8	0.203	0.0259	ns	****
	HepaRG static (standard) wk4	3.188	0.1915	****	
	HepaRG static (standard) wk8	3.476	0.1296	****	
	HepaRG static (HEPES) wk4	0.771	0.0831	****	****
	HepaRG static (HEPES) wk8	0.464	0.0358	ns	****
	Primary human hepatocytes	0.274	0.0185		
**CYP2C8**	HepaRG flow (HEPES) wk4	0.332	0.1364	*	****
	HepaRG flow (HEPES) wk8	0.515	0.2176	ns	**
	HepaRG static (standard) wk4	1.965	0.0991	**	
	HepaRG static (standard) wk8	2.637	0.6876	****	
	HepaRG static (HEPES) wk4	0.820	0.1089	ns	***
	HepaRG static (HEPES) wk8	0.827	0.0396	ns	*
	Primary human hepatocytes	1.062	0.0759		
**CYP2C9**	HepaRG flow (HEPES) wk4	0.459	0.2738	****	*
	HepaRG flow (HEPES) wk8	0.987	0.4523	****	**
	HepaRG static (standard) wk4	1.217	0.2603	***	
	HepaRG static (standard) wk8	3.303	0.1944	**	
	HepaRG static (HEPES) wk4	0.584	0.0806	****	*
	HepaRG static (HEPES) wk8	0.407	0.0249	****	****
	Primary human hepatocytes	2.384	0.1710		
**CYP2C18**	HepaRG flow (HEPES) wk4	1.122	0.6752	****	ns
	HepaRG flow (HEPES) wk8	2.238	1.0022	****	*
	HepaRG static (standard) wk4	0.173	0.0144	****	
	HepaRG static (standard) wk8	0.423	0.0727	****	

	HepaRG static (HEPES) wk4	0.075	0.0025	****	***
	HepaRG static (HEPES) wk8	0.097	0.0107	****	**
	Primary human hepatocytes	4.977	0.2122		
**CYP2C19**	HepaRG flow (HEPES) wk4	0.791	0.0276	ns	*
	HepaRG flow (HEPES) wk8	0.880	0.1786	ns	*
	HepaRG static (standard) wk4	1.736	0.5704	**	
	HepaRG static (standard) wk8	1.286	0.1491	ns	
	HepaRG static (HEPES) wk4	1.054	0.2545	ns	ns
	HepaRG static (HEPES) wk8	0.944	0.0798	ns	*
	Primary human hepatocytes	0.685	0.0793		
**CYP2E1**	HepaRG flow (HEPES) wk4	0.522	0.2596	****	**
	HepaRG flow (HEPES) wk8	0.722	0.2478	**	***
	HepaRG static (standard) wk4	1.644	0.1771	ns	
	HepaRG static (standard) wk8	3.058	0.1850	****	
	HepaRG static (HEPES) wk4	0.125	0.0440	****	***
	HepaRG static (HEPES) wk8	0.206	0.0268	****	*****
	Primary human hepatocytes	1.425	0.0646		
**CYP2J2**	HepaRG flow (HEPES) wk4	0.930	0.4137	****	ns
	HepaRG flow (HEPES) wk8	1.434	0.4832	**	ns
	HepaRG static (standard) wk4	1.017	0.0569	****	
	HepaRG static (standard) wk8	1.554	0.1228	**	
	HepaRG static (HEPES) wk4	0.577	0.0686	****	**
	HepaRG static (HEPES) wk8	0.508	0.0348	****	***
	Primary human hepatocytes	2.525	0.2965		
**CYP3A4**	HepaRG flow (HEPES) wk4	0.165	0.0184	ns	***
	HepaRG flow (HEPES) wk8	0.288	0.0903	ns	*
	HepaRG static (standard) wk4	1.625	0.2457	****	
	HepaRG static (standard) wk8	1.439	0.5551	***	
	HepaRG static (HEPES) wk4	1.911	0.3372	****	ns
	HepaRG static (HEPES) wk8	1.240	0.0547	**	ns
	Primary human hepatocytes	0.170	0.0081		
**CYP3A5**	HepaRG flow (HEPES) wk4	0.631	0.1656	***	ns
	HepaRG flow (HEPES) wk8	0.958	0.3872	**	**
	HepaRG static (standard) wk4	0.863	0.0799	**	
	HepaRG static (standard) wk8	2.104	0.1737	ns	
	HepaRG static (HEPES) wk4	1.111	0.2804	ns	ns
	HepaRG static (HEPES) wk8	1.215	0.1228	ns	**
	Primary human hepatocytes	1.631	0.0871		
**CYP3A7**	HepaRG flow (HEPES) wk4	0.273	0.0967	ns	***
	HepaRG flow (HEPES) wk8	0.309	0.1090	ns	**
	HepaRG static (standard) wk4	1.340	0.1365	ns	
	HepaRG static (standard) wk8	4.254	1.3311	****	
	HepaRG static (HEPES) wk4	1.222	0.2478	ns	ns
	HepaRG static (HEPES) wk8	1.153	0.1628	ns	*
	Primary human hepatocytes	0.476	0.0260		
	**Transport towards bile canuliculi**				
**ABCB1 (PGP)**	HepaRG flow (HEPES) wk4	0.406	0.0871	ns	****
	HepaRG flow (HEPES) wk8	0.488	0.1249	ns	***
	HepaRG static (standard) wk4	2.072	0.1415	****	
	HepaRG static (standard) wk8	1.404	0.0852	****	
	HepaRG static (HEPES) wk4	1.915	0.1947	****	ns
	HepaRG static (HEPES) wk8	1.165	0.0518	***	*

	Primary human hepatocytes	0.684	0.0387		
ABCB4 (MDR3)	HepaRG flow (HEPES) wk4	0.441	0.0271	ns	****
	HepaRG flow (HEPES) wk8	0.418	0.1032	ns	****
	HepaRG static (standard) wk4	1.766	0.1004	****	
	HepaRG static (standard) wk8	3.379	0.3092	****	
	HepaRG static (HEPES) wk4	0.803	0.1432	ns	***
	HepaRG static (HEPES) wk8	0.727	0.0455	ns	***
	Primary human hepatocytes	0.636	0.0424		
ABCB11 (BSEP)	HepaRG flow (HEPES) wk4	1.009	0.5742	***	ns
	HepaRG flow (HEPES) wk8	1.401	0.5591	**	ns
	HepaRG static (standard) wk4	0.853	0.1894	****	
	HepaRG static (standard) wk8	1.624	0.4528	**	
	HepaRG static (HEPES) wk4	0.106	0.0399	****	**
	HepaRG static (HEPES) wk8	0.291	0.0328	****	**
	Primary human hepatocytes	2.793	0.1318		
ABCC2 (MRP2)	HepaRG flow (HEPES) wk4	0.615	0.1769	***	***
	HepaRG flow (HEPES) wk8	0.931	0.3351	**	*
	HepaRG static (standard) wk4	2.132	0.1997	****	
	HepaRG static (standard) wk8	1.631	0.2223	**	
	HepaRG static (HEPES) wk4	1.454	0.1183	****	**
	HepaRG static (HEPES) wk8	0.754	0.0347	****	**
	Primary human hepatocytes	1.653	0.0300		
ABCG2 (BCRP)	HepaRG flow (HEPES) wk4	0.917	0.3195	**	ns
	HepaRG flow (HEPES) wk8	1.120	0.4053	*	ns
	HepaRG static (standard) wk4	1.231	0.2062	ns	
	HepaRG static (standard) wk8	0.724	0.1581	***	
	HepaRG static (HEPES) wk4	1.096	0.2670	*	ns
	HepaRG static (HEPES) wk8	0.708	0.0413	***	ns
	Primary human hepatocytes	1.836	0.0776		
SLC47A1 (MATE)	HepaRG flow (HEPES) wk4	0.333	0.0255	ns	****
	HepaRG flow (HEPES) wk8	0.365	0.0173	ns	***
	HepaRG static (standard) wk4	1.456	0.0411	****	
	HepaRG static (standard) wk8	1.839	0.1759	****	
	HepaRG static (HEPES) wk4	1.678	0.3249	****	ns
	HepaRG static (HEPES) wk8	1.378	0.0632	****	*
	Primary human hepatocytes	0.249	0.0189		
	**Transport in/out of the hepatocyte**				
NTCP	HepaRG flow (HEPES) wk4	0.859	0.4598	****	ns
	HepaRG flow (HEPES) wk8	1.293	0.4638	***	ns
	HepaRG static (standard) wk4	0.445	0.0141	****	
	HepaRG static (standard) wk8	1.057	0.1449	***	
	HepaRG static (HEPES) wk4	0.387	0.1005	****	ns
	HepaRG static (HEPES) wk8	0.695	0.0411	****	*
	Primary human hepatocytes	2.378	0.0692		
SLCO1B1	HepaRG flow (HEPES) wk4	1.240	0.7494	****	ns
	HepaRG flow (HEPES) wk8	2.051	0.8809	***	*
	HepaRG static (standard) wk4	0.191	0.0049	****	
	HepaRG static (standard) wk8	0.181	0.0285	****	
	HepaRG static (HEPES) wk4	0.092	0.0306	****	**
	HepaRG static (HEPES) wk8	0.085	0.0085	****	**
	Primary human hepatocytes	4.310	0.2567		

SLCO1B3	HepaRG flow (HEPES) wk4	1.210	0.3202	****	*
	HepaRG flow (HEPES) wk8	1.840	0.5952	***	*
	HepaRG static (standard) wk4	0.374	0.0366	****	
	HepaRG static (standard) wk8	0.439	0.0850	****	
	HepaRG static (HEPES) wk4	0.367	0.0106	****	ns
	HepaRG static (HEPES) wk8	0.418	0.0109	****	ns
	Primary human hepatocytes	3.076	0.2101		
SLCO2B1	HepaRG flow (HEPES) wk4	0.327	0.1509	**	***
	HepaRG flow (HEPES) wk8	0.468	0.1537	*	****
	HepaRG static (standard) wk4	1.431	0.0903	**	
	HepaRG static (standard) wk8	2.956	0.1528	****	
	HepaRG static (HEPES) wk4	0.880	0.2663	ns	*
	HepaRG static (HEPES) wk8	1.029	0.1066	ns	****
	Primary human hepatocytes	0.929	0.0323		
SLC22A1 (OCT1)	HepaRG flow (HEPES) wk4	0.589	0.3041	**	**
	HepaRG flow (HEPES) wk8	1.009	0.3669	ns	*
	HepaRG static (standard) wk4	1.884	0.0906	ns	
	HepaRG static (standard) wk8	2.016	0.4928	ns	
	HepaRG static (HEPES) wk4	0.635	0.1316	**	***
	HepaRG static (HEPES) wk8	0.848	0.0958	*	*
	Primary human hepatocytes	1.696	0.1618		
ABCC3 (MRP3)	HepaRG flow (HEPES) wk4	0.273	0.1259	ns	**
	HepaRG flow (HEPES) wk8	0.392	0.1214	ns	**
	HepaRG static (standard) wk4	1.736	0.4126	*	
	HepaRG static (standard) wk8	2.855	0.6221	****	
	HepaRG static (HEPES) wk4	0.706	0.1118	ns	*
	HepaRG static (HEPES) wk8	0.703	0.1516	ns	**
	Primary human hepatocytes	0.816	0.1522		
	**Nuclear transcription**				
AHR	HepaRG flow (HEPES) wk4	0.898	0.0651	ns	**
	HepaRG flow (HEPES) wk8	0.918	0.0608	ns	ns
	HepaRG static (standard) wk4	1.429	0.1180	****	
	HepaRG static (standard) wk8	1.073	0.1280	**	
	HepaRG static (HEPES) wk4	1.276	0.0669	****	ns
	HepaRG static (HEPES) wk8	0.854	0.0418	ns	*
	Primary human hepatocytes	0.780	0.0834		
NR1L2 (PXR)	HepaRG flow (HEPES) wk4	0.570	0.2755	***	ns
	HepaRG flow (HEPES) wk8	0.782	0.2648	**	**
	HepaRG static (standard) wk4	0.918	0.0547	*	
	HepaRG static (standard) wk8	2.162	0.0871	***	
	HepaRG static (HEPES) wk4	0.999	0.1118	ns	ns
	HepaRG static (HEPES) wk8	1.158	0.0251	ns	****
	Primary human hepatocytes	1.337	0.1302		
	**Hepatocyte marker**				
ALB	HepaRG flow (HEPES) wk4	0.473	0.2064	****	**
	HepaRG flow (HEPES) wk8	0.727	0.2925	***	**
	HepaRG static (standard) wk4	1.607	0.2709	ns	
	HepaRG static (standard) wk8	2.198	0.0821	*	
	HepaRG static (HEPES) wk4	0.918	0.1500	**	*
	HepaRG static (HEPES) wk8	1.027	0.0190	**	****
	Primary human hepatocytes	1.659	0.0681		

Relative mRNA expression (against the expression level of the housekeeping genes GAPDH, RPL27 and 36B3) of 28 genes involved in chemical biotransformation (CYPs) and transport in HepaRG cells that were either cultured under flow conditions or static conditions (with either standard culture medium or HEPES-buffered medium) for 4 weeks and 8 weeks, and in pooled human primary hepatocytes. For each gene, the expression was compared with the expression in primary hepatocytes, and with the expression in static HepaRG cells under standard conditions within each week. * $P<0.05$; ** $P<0.01$; *** $P<0.001$; **** $P<0.0001$; ns. not significant (One-way ANOVA and Bonferroni post-hoc tests).

Supplementary table 2 *Primer sequences*

Gene Symbol	Forward primer 5’-> 3’	Reverse primer 5’-> 3’
CYP1A1	TCGGCCACGGAGTTTCTTC	GGTCAGCATGTGCCCAATCA
CYP1A2	ATGCTCAGCCTCGTGAAGAAC	GTTAGGCAGGTAGCGAAGGAT
CYP1B1	ACGTACCGGCCACTATCACT	CTCCCCACGACCTGATCCA
CYP2B6	CAGCCACCAGAACCTCAACC	AAGGTCGGAAAATCTCTGAATCTCATA
CYP2C18	GGAAAACGGATGTGTATGGGAG	GTGGCACACGACCAAATGC
Cyp2C19	ATTGAATGAAAACATCAGGATTG	GAGGGTTGTTGATGTCCATC
CYP2C8	GGACTTTATCGATTGCTTCCTG	CCATATCTCAGAGTGGTTGCTTG
CYP2C9	CCTCTGGGGCATTATCCATC	ATATTTGCACAGTGAAACATAGGA
CYP2E1	GTGATGCACGGCTACAAGG	GGGTGGTCAGGGAAAACCG
CYP2J2	TGGCTTGCCCTTAATCAAAGAA	GGCCACTTGACATAATCAATCCA
CYP3A4	AAGTCGCCTCGAAGATACACA	AAGGAGAGAACACTGCTCGTG
CYP3A5	AATGTTTTGTCCTATCGTCAGGG	AGACCTTCGATTTGTGAAGACAG
CYP3A7	AAACTTGGCCGTGGAAACCT	CCTTACGGAAGGACAAAGCATTT
AHR	CAAATCCTTCCAAGCGGCATA	CGCTGAGCCTAAGAACTGAAAG
NR1L2 (PXR)	GCCCATGCTGAAATTCCACTA	GCCGATTGCATTCAATGTAGGA
ALB	GGAAAAGTGGGCAGCAAATGT	GGTTCAGGACCACGGATAGA
ABCB1 (PGP)	TTGCTGCTTACATTCAGGTTTCA	AGCCTATCTCCTGTCGCATTA
ABCB4 (MDR3)	ATAGCTCACGGATCAGGTCTC	GGATTTAGCAGCGACAAGGAAA
ABCB11 (BSEP)	GCCGCAGCTCGTCAGATAC	GAATTGCAGTCAAACCACCCTAT
ABCC2 (MRP2)	TCTCTCGATACTCTGTGGCAC	CTGGAATCCGTAGGAGATGAAGA
ABCG2 (BCRP)	ACGAACGGATTAACAGGGTCA	CTCCAGACACACCACGGAT
SLC47A1 (MATE1)	TCAACCAGGGAATTGTACTGC	CAGAGCCTATCACCCCAAGA
ABCC3 (MRP3)	TGGGGTGAAGTTTCGTACTGG	CACGTTTGACTGAGTTGGTGATA
NTCP	CATAGGGATCGTCCTCAAATCCA	GCCACACTGCACAAGAGAATG
SLC22A1 (OCT1)	TGTCACCGAAAAGCTGAGCC	TCCGTGAACCACAGGTACATC
SLCO1B1 (OATP1B1)	TTGGAGGTGTTTTGACTGCTT	ACAAGTGGATAAGGTCGATGTTG
SLCO1B3 (OATP1B3)	GTCCAGTCATTGGCTTTGCA	CAACCCAACGAGAGTCCTTAGG
SLCO2B1 (OATP2B1)	GGCAAGGACTCTCCCTCTAAG	GTTTGGTGCAATCTGGACTAGG
36B4 *	CGGGAAGGCTGTGGTGCTG	GTGAACACAAAGCCCACATTCC
GAPDH *	CTCTGCTCCTCCTGTTCGAC	TTAAAAGCAGCCCTGGTGAC
RPL27 *	ATCGCCAAGAGATCAAAGATAA	TCTGAAGACATCCTTATTGACG

Primer sequences of the primers used for quantitative (RT-qPCR). Genes denoted with an asterisk were used as reference genes.
